# Atypical Serogroup IVb-v1 of *Listeria monocytogenes* Assigned to New ST2801, Widely Spread and Persistent in the Environment of a Pork-Meat Producing Plant of Central Italy

**DOI:** 10.3389/fmicb.2022.930895

**Published:** 2022-06-27

**Authors:** Fabrizia Guidi, Cinzia Lorenzetti, Gabriella Centorotola, Marina Torresi, Cesare Cammà, Alexandra Chiaverini, Francesco Pomilio, Giuliana Blasi

**Affiliations:** ^1^Istituto Zooprofilattico Sperimentale dell’Umbria e delle Marche “Togo Rosati,” Perugia, Italy; ^2^Laboratorio Nazionale di Riferimento per Listeria Monocytogenes, Istituto Zooprofilattico Sperimentale dell’Abruzzo e del Molise G. Caporale, Teramo, Italy; ^3^Centro di Referenza Nazionale per Sequenze Genomiche di Microrganismi Patogeni, Istituto Zooprofilattico Sperimentale dell’Abruzzo e del Molise G. Caporale, Teramo, Italy

**Keywords:** *Listeria monocytogenes*, serogroup IVb-v1, ST2801 (CC218), food producing environment, persistence, WGS typing, environmental sampling

## Abstract

In this study, we characterized 84 *Listeria monocytogenes* (Lm) strains having an atypical IVb-v1 profile and isolated in a meat producing plant of Central Italy. They were assigned to the new MLST type ST2801 (CC218). The new ST was widespread in the food-producing environment where it was able to persist for over a year even after cleaning and sanitation. Cluster analysis identified three main clusters genetically close to each other (0–22 allelic differences and 0–28 SNPs) from two different cgMLST types, suggesting a common source. The coexistence of closely related clusters over time could be the result of a different evolution path starting from a common ancestor first introduced in the plant and/or the consequence of the repetitive reintroduction of closely related clones probably by raw materials. All the strains presented several determinants for heavy metals resistance, stress response, biofilm production, and multidrug efflux pumps with no significant differences among the clusters. A total of 53 strains carried pLI100 and the j1776 plasmids, while in one strain, the pLM33 was found in addition to pLI100. Only the strains carrying plasmids presented *cadA* and *cadC* for cadmium resistance and the *mco* gene encoding a multicopper oxidase and *gerN* for an additional Na+/H+-K+ antiporter. All the strains presented a virulence profile including a full-length *inlA* gene and the additional LIPI-3. The isolation of a new ST with a large pattern of stress-adaptation genes and able to persist is an important contribution to deepening the current knowledge on the uncommon IVb-v1 and in general on the genomic diversity of Lm.

## Introduction

*Listeria monocytogenes* (Lm) is the foodborne pathogen causing human listeriosis, the most serious foodborne disease under EU surveillance with the highest proportion of hospitalized cases and fatality rate (13%). Invasive forms of the disease mainly affect people at risk causing abortion and stillbirth in pregnant women and meningitis septicemia and death in the elderly, immunocompromised people, and newborns (European Food Safety Authority, and European Centre for Disease Prevention and Control, 2021). Lm is a genetically heterogeneous species including hypo- and hypervirulent clones (Maury et al., 2016, 2019) and strains able to survive and persist in food-producing environments (FPE) even for years, due to their adaptation to different environmental stresses such as cold temperatures, high salinity, low pH, oxidation, and desiccation (Palma et al., 2017; Pombinho et al., 2017; Maury et al., 2019; Parsons et al., 2020; Guidi et al., 2021; Palaiodimou et al., 2021).

*Listeria monocytogenes* isolates can be grouped into four lineages, five most common PCR serogroups (Orsi et al., 2011), 2,880 multilocus sequence typing (MLST) sequence types (ST) grouping in different clonal complexes (CCs) (Ragon et al., 2008), and thousands of core genome MLST (cgMLST) types (CTs) grouped into nearly 400 different sublineages (Moura et al., 2017) (accessed on February 2022).^1^

Although the most common Lm PCR serogroups known are IIa, IIb, IIc, L, and IVb (Doumith et al., 2004; Kérouanton et al., 2010; Leclercq et al., 2011), an atypical and novel PCR profile of serogroup IVb was identified in 2007 for Lm isolates from France (Leclercq et al., 2011) and other countries including the United States (Graves et al., 2007), Chile (de Vasconcelos et al., 2008), and Australia (Huang et al., 2011). These atypical strains harbored the *lmo0737* gene, specific to serogroups IIa and IIc in addition to the four-target profile defining serogroup IVb (Doumith et al., 2004; Kérouanton et al., 2010; Leclercq et al., 2011; Lee et al., 2012). This rare profile was designated as “IVb-v1” (Lee et al., 2012).

Although the first studies published on this atypical IVb-v1 profile reported its isolation from milk products and meat products (Leclercq et al., 2011), the most recent works show a particular association with vegetable matrices such as caramel apple, stone fruits, leafy green, and radicchio as well as the associated processing environments (Torresi et al., 2020; Yang et al., 2020; Chen et al., 2022). Scientific reports on different IVb-v1 clinical isolates are also available (Leclercq et al., 2011; Lee et al., 2012; Scaltriti et al., 2020).

All the IVb-v1 strains previously isolated worldwide, from both food and humans, mainly belonged to four STs: ST218 (CC218), ST240 (CC240), ST382 (CC183), and ST554 (CC554) (Chen et al., 2017, 2022; Kim et al., 2018; Scaltriti et al., 2020; Yang et al., 2020).

To date, very few in-depth studies have been performed on the virulence genetic profile of Lm strains belonging to IVb-v1 and their genetic determinants involved in stress response. Chen et al. (2022), reported the presence of different stress response determinants in Lm IVb-v1 belonging to ST554 (low pH, cold, etc.) in the only published study describing the persistence of IVb-v1 strains in a food-processing plant. The same authors also defined the virulence profile for internalins and *Listeria* Pathogenicity Islands (LIPI) identifying the presence of *inlA/B/C/E/F/H/J/K/P* and the LIPI-3. The latter was also reported by de Tavares et al. (2020) in IVb-v1 Lm isolates belonging to ST218. In their report on a IVb-v1 Lm isolated from a vegetable matrix, Torresi et al. (2020) described the presence of a plasmid which carried heavy metal-resistance genes, but no information about the virulence profile was provided.

During the extensive environmental sampling plan for Lm performed in a pork meat-producing plant of Central Italy between 2020 and 2021, we isolated several strains having the atypical IVb-v1 serogroup profile and belonging to a new ST assigned by the Institute Pasteur (ST2801).

In this study, we characterized the new ST2801 isolates to (i) evaluate the genomic correlation existing among them, (ii) assess their persistence in the meat producing plant, (iii) investigate the presence of genetic determinants involved in environmental stress adaptation, and (iv) define virulence profiles.

## Materials and Methods

### Environmental Sampling and *Listeria monocytogenes* Detection

During the period between July 2020 and September 2021, an extensive environmental sampling for Lm was performed in a pork meat-producing plant in Central Italy. Three different sampling sessions were performed during production, in particular, on July 2020 (Production 1), May 2021 (Production 2), and September 2021 (Production 3). A sampling scheme of 63 surfaces including both food contact (FCS) and nonfood contact surfaces (NFCS) was defined focusing on the main niches for Lm presence (Supplementary Table 1). In each sampling session, the same surfaces were sampled using commercial sterile sponges.

In accordance with the European Union Reference Laboratory for Lm (EURL) guidelines (Carpentier and Barre, 2012), the total sampled area varied depending on the sampling site but was as large as possible to improve the probability of detecting Lm. The samples were tested according to ISO 11290-1:2017 for Lm detection.

If positive surfaces were found, they were sampled again after extraordinary cleaning and sanitation.

### Strains Collection

Up to five Lm colonies from each positive sample were randomly selected and screened for their belonging to one of the five major serogroups (IIa, IIb, IIc, L, and IVb), using a multiplex PCR assay according to the EURL method (Doumith et al., 2004; Kérouanton et al., 2010). At least one isolate for each serogroup found in each sample was selected to be subjected to whole genome sequencing (WGS). In this study, we focused on Lm strains presenting the atypical serogroup IVb-v1 (Lee et al., 2012).

### Whole Genome Sequencing and Bioinformatic Analysis

DNA extraction was performed according to Portmann et al. (2018), with minor modifications, using the QIAamp DNA Mini Kit (Qiagen Hilden, Germany) according to the manufacturer’s protocol.

The purity of the extracts was evaluated using NanoDrop2000 (ThermoFisher Scientific, Wältham, MA, United States). Starting from 1 ng of input DNA, the Nextera XT DNA chemistry (Illumina, San Diego, CA, United States) for library preparation was used according to the manufacturer’s protocols. WGS was performed on the NextSeq 500 platform (Illumina, San Diego, CA, United States) with the NextSeq 500/550 mid output reagent cartridge v2 (300 cycles, standard 150-bp paired-end reads).

For the analysis of WGS data, an in-house pipeline (Cito et al., 2018) was used, which included steps for trimming (Trimmomatic version 0.36^2^; base quality parameters, namely, leading, 25; trailing, 25; and sliding window, 20:25) (Bolger et al., 2014) and a quality control check of the reads (FastQC version 0.11.5^3^).

Genome *de novo* assembly of paired-end reads was performed using SPAdes version 3.11.1^4^ (Bankevich et al., 2012) with the parameters suggested by the manual for the Illumina platform 2_150 chemistry (--only-assembler --careful -k 21, 33, 55, 77). Then, the genome assembly quality check was performed using QUAST version 4.3^5^ (Gurevich et al., 2013). All the genomes that met the quality parameters recommended by Timme et al. (2020) were used for the subsequent analysis steps.

The genome assemblies were deposited at DDBJ/ENA/GenBank under the BioProject PRJNA821663.

### Multilocus Sequence Typing Analysis, Core Genome Multilocus Sequence Typing, and Single-Nucleotide Polymorphisms Analysis

The sequence type (ST) and the clonal complex (CC) were deducted *in silico* using the specific tool available on the BIGSdb-Lm database (accessed on October 2021)^6^ and based on the MLST scheme including the seven housekeeping genes *abcZ*, *bglA*, *cat*, *dapE*, *dat*, *ldh*, and *lhlA* (Ragon et al., 2008).

To verify the relatedness among the isolates, identifying genomic clusters, a cgMLST analysis was performed using the chewBBACA^7^ allele calling algorithm (Silva et al., 2018) and the Pasteur Institute cgMLST scheme of 1,748 loci (Moura et al., 2017). According to the guidelines for Lm cgMLST typing (Moura et al., 2017), only the genomes with at least 1,660 called loci (95% of the full scheme) were considered. The software GrapeTree^8^ (Zhou et al., 2018) was used for the visualization of the minimum spanning tree (MSTreeV2 method).

A core single-nucleotide polymorphism (SNPs) analysis was performed using the reference-free tool KSNP3^9^ with a kmer size of 21 (Morganti et al., 2016). The resulting neighbor-joining (NJ) tree was visualized using the interactive tree of life (iTOL).^10^

### Genetic Determinants Involved in Stress Adaptation, Biofilm Formation, and Virulence Potential

All the genome assemblies were manually screened for the presence/absence of loci encoding for disinfectants and metal resistance and stress survival islets (SSIs) using the “Metal and Detergent Resistance” and the “Stress Islands” tools available on the BIGSdb-Lm platform (accessed on January 2022).

Moreover, the detection of additional determinants in the field, not included in these schemes (*sugE*, *mdrl*, *lde*, *arsRDABC*, *cadAC*), was performed automatically using Prokka v.1.12^11^ (Seemann, 2014).

Genes involved in biofilm production were also detected using the BIGSdb-Lm platform (*inlA*, *actA*, *prfA*, *lmo0673*, and *lmo2504*) and the Prokka software (see text footnote 11) (*luxS*, *recO*, *inlL*, and *bapL*).

The PlasmidFinder web tool (version 2.0.1 2020-02-07; accessed from October 2021 to January 2022; Carattoli et al., 2014)^12^ was used to detect the presence of presumptive plasmids.

The virulence profile of the strains was deduced using the “Virulence” tool provided by the BIGSdb-Lm platform, also investigating the presence of premature stop codon mutations in the *inlA* gene (accessed from October 2021 to January 2022).

## Results

### Environmental Sampling and *Listeria monocytogenes* Strain Collection

During the period between July 2020 and September 2021, 189 environmental samples were collected in the pork meat-producing plant of the study during production and 40 after cleaning and sanitation. A total of 35 samples of those collected during production (18.5%) and 7 samples of those collected after cleaning and sanitation (17.5%) tested positive for the presence of Lm. A total of 147 Lm strains were isolated from these samples and screened for serogroup. Among them, 84 Lm strains (Supplementary Table 2), isolated from 19 environmental surfaces, presented the atypical IVb-v1 serogroup profile carrying the *lmo0737* target gene in addition to the four targets normally characterizing the serogroup IVb (*ORF2110, ORF2819*, *prs* and *prfA*). These atypical strains were selected for further genomic analysis.

### Multilocus Sequence Typing Analysis, Core Genome Multilocus Sequence Typing, and Single-Nucleotide Polymorphisms Analysis

For all the Lm strains IVb-v1, the MLST analysis found exact allele matches for only six of the seven genes of the scheme. After submitting the genomes to the BIGSdb-Lm database (21 October 2021), a new allelic id was defined for the locus *abcZ* resulting in a new MLST profile (named ST2801 and belonging to clonal complex CC218).

**FIGURE 1:**
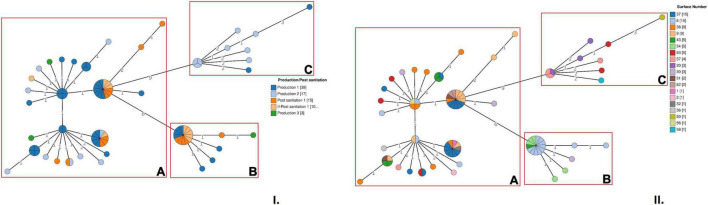
Cluster analysis of Lm strains belonging to ST2801 based on cgMLST profiles. **(I)** In the minimum spanning tree (MSTv2), strains are colored according to the sampling session. **(II)** In the MSTv2, strains are colored according to the environmental surface from which they were isolated. The number values between adjacent nodes indicate the number of allelic differences between nodes. Clusters are highlighted with red boxes.

The cgMLST analysis showed that the microbial population associated with the new ST was heterogeneous and allelic differences among the strains ranged from 0 to 22 (Figure 1). Based on a seven allele threshold, three main clusters were identified (Figure 1). Cluster A (cgMLST type L1-SL218-ST2801-CT10448) included 56 strains and was isolated in the FPE after all the sampling sessions performed during production and after two consecutive cleaning and sanitation sessions subsequent to Production 1. This cluster was isolated from 15 different surfaces: n°1, 2, 6, 9, 30, 32, 36, 37, 38, 43, 51, 56, 57, 62, and 63 (Supplementary Table 2). Among these surfaces, n°9, 30, 37, 38, and 63 were found to be contaminated with cluster A more than once over the course of time.

A total of 18 Lm strains, isolated during Production 1 and Production 3 and after two consecutive cleaning and sanitation sessions subsequent to Production 1 (I-Post sanitation 1 and II-Post sanitation 1), were grouped in cluster B (cgMLST type L1-SL218-ST2801-CT10448). No strains isolated during Production 2 were grouped in this cluster. A total of 4 surfaces of the scheme, n°6, 30, 43 and 54, were found to be contaminated with cluster B, with surface n°6 resulting positive more than once (Figure 1 and Supplementary Table 2).

Finally, cluster C (cgMLST type L1-SL218-ST2801-CT11418) included 10 strains all isolated during production, in particular, Production 1 and Production 2. This cluster was recovered from 5 different surfaces of the sampling scheme, n°20, 50, 57, 58, and 63 (Figure 1).

**FIGURE 2:**
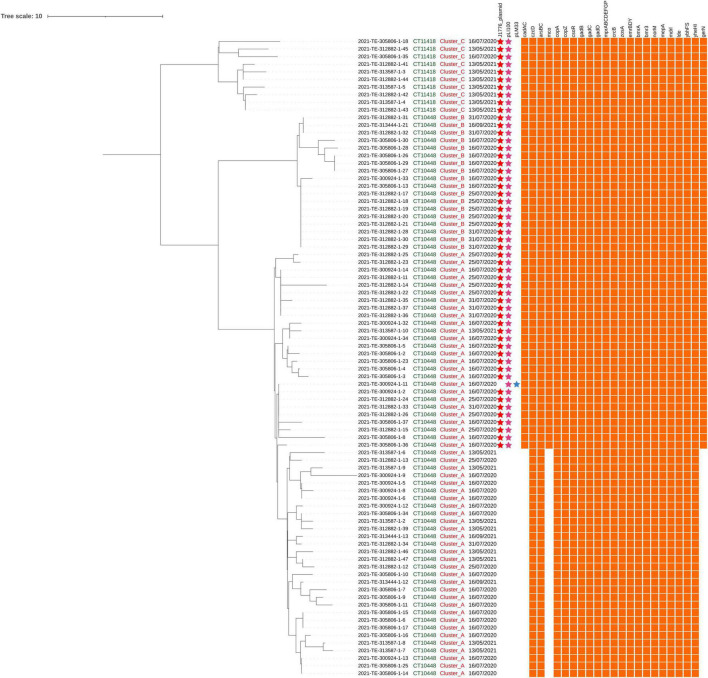
Single-nucleotide polymorphisms (SNPs) analysis of Lm strains belonging to ST2801. The first, the second, and the third columns indicate the CT, the cluster, and the sampling date, respectively. The presence/absence matrix represents, from left to right, plasmids (j1776 plasmid, pLI100, and pLM33) and genes involved in stresses adaptation.

The core SNPs analysis was performed to deepen the genetic relationships between the strains and the results were concordant with those of the cgMLST analysis (Figure 2). The number of SNP differences among all the isolates ranged from 0 to 28 (Supplementary Table 3). In particular, Lm strains grouped in cluster A differed by a number of SNPs ranging from 0 to 20 with a median of 4. Among strains of cluster B and cluster C, SNPs differences ranged from 0 to 5 (median 1) and from 0 to 11 (median 6), respectively.

### Genetic Determinants Involved in Stress Adaptation and Biofilm Production

Using the BIGSdb-Lm platform together with the annotation results, several determinants for different heavy metal resistance were detected, as well as genes for multidrug efflux pumps and response to environmental stresses (Table 1).

**TABLE 1:** Relevant features for environmental persistence of different clusters conveyed and not by plasmids.

Main function		Gene	Localization	References
Metal resistance	Cadmium	cadA	J1776plasmid	Parsons et al., 2018
		cadC	pLI100	Parsons et al., 2018
		czcD	Chromosome	Osman et al., 2021
	Arsenic	arsB, arsC	Chromosome	Parsons et al., 2018
	Copper	copA, copZ, csoR	Chromosome	Corbett et al., 2011
		mco	J1776plasmid	Schmitz-Esser et al., 2021
	Zinc	czcD	Chromosome	Osman et al., 2021
Stress response	Acid tolerance	gadB, gadC, gadD_SSI1	Chromosome	Cotter et al., 2005; Ryan et al., 2010; Liu et al., 2019
	Alkali response	mprA, mrpB mprC, mrpD, mrpE, mrpF, mprG, mdrP	Chromosome	Abdel-Motaal et al., 2018; Fang et al., 2018; Xu et al., 2018; Yan et al., 2022
	Fluorides	crcB	Chromosome	Baker et al., 2012; Johnston and Strobel, 2020; Chellaiah et al., 2021
	Oxidative response	zosA	Chromosome	Gaballa and Helmann, 2002
	Saline response	gerN	pLI100	Southworth et al., 2001; Wu et al., 2020
Biocides resistance	Multidrug efflux-pumps	emrB, emrD, emrY, bmrA, bmr3, norM, mepA, mdrl, lde	Chromosome	Slipski et al., 2018; Sharma et al., 2019; Cherifi et al., 2020; Matereke and Okoh, 2020; Zhang et al., 2020
	Multidrug ABC transporter	ybhF, ybhS, yheI, yheH	Chromosome	Torres et al., 2009; Feng et al., 2020
Biofilm production		luxS, recO, full length actA, lmo0673, lmo2504	Chromosome	Pasquali et al., 2018; Gorski et al., 2022

In all the strains, the determinants for heavy metal resistance included *czcD*, *arsB*, *arsC*, and the *csoR-copA-copZ* copper-resistance operon. The *zosA* gene for Zn(II) uptake was also found.

The same genetic determinants for stress responses were detected in all the strains. In particular, for tolerance to acid stress, in addition to the *gadB-gadC* operon, *gadD*, was also found in all the strains and represented the only gene of the SSI-1.

Several determinants associated with monovalent cation/proton antiporters were found in all the ST2801 strains. These genes included the multiple resistance and pH operons *mrpABCDEFG* and *mdrP*.

All the strains of the new ST carried determinants for different multidrug efflux pumps (*emrB*, *emrD*, *emrY*, *bmrA*, *bmr3*, *norM*, *mepA, mdrl*, and *lde*) and multidrug ABC transporters (*ybhF*, *ybhS*, *yheI*, and *yheH*), as well as the fluoride resistance gene *crcB*.

A total of 26 strains from cluster A and all the strains from cluster B and cluster C carried two different plasmids and in particular, the pLI100 (Acc. Number AL592102) together with the j1776 plasmid (Acc. Number CP006612) or the pLM33 (Acc. Number GU244485; Figure 2).

Only the strains that carried these plasmids presented *cadA* and *cadC* for cadmium resistance, the *mco* gene for copper detoxification and the additional Na+/H+-K+ antiporter GerN.

All the strains carried the genes *luxS*, *recO*, *lmo2504*, and *lmo0673* involved in biofilm formation.

### Virulence Genes

A total of 66 virulence genes were detected on a scheme of 93 targets. All the strains showed the same virulence profile that presented the Listeria Pathogenicity Island (LIPI) 3 (*llsA*, *llsG*, *llsH*, *llsX*, *llsB*, *llsY*, *llsD*, and *llsP*) in addition to the conventional LIPI-1 (*prfA*, *actA*, *hly*, *mpl*, *plcA*, *plcB*, and *iap*), 10 internalin genes including a full length *inlA*, *inlB*, *inlC*, *inlD*, *inlE*, *inlF*, *inlH*, *inlJ*, and *inlK*, the v*iR/virS* virulence regulatory system, the teichoic acid biosynthesis genes *gltA* and *gltB*, and the invasion gene *aut_IVb*.

## Discussion

In this study, we reported the widespread isolation of Lm strains having an atypical IVb-v1 serogroup profile from the FPE of a meat-producing plant in Central Italy. To date, very little is known about the circulation of this uncommon variant in Italy, where only two studies reported the isolation of Lm strain IVb-v1 (Scaltriti et al., 2020; Torresi et al., 2020).

The first studies published by authors of other countries on this atypical IVb-v1 profile reported its isolation from milk products and meat products (Leclercq et al., 2011). However, the most recent works, including the one of Torresi et al. (2020), also show the association with vegetable matrices such as caramel apple, stone fruits, leafy green, and radicchio (Torresi et al., 2020; Yang et al., 2020; Chen et al., 2022). Moreover, IVb-v1 Lm strains have been previously isolated from FPEs. In more detail, Lee et al. (2012) typed three IVb-v1 strains isolated from a turkey processing plant of the United States during a 2-year sampling performed by Mullapudi et al. (2008) between 2017 and 2018. These strains were isolated from two drains and a chiller re-work table.

Chen et al. (2022) used WGS to type IVb-v1 strains isolated from several environmental surfaces of an apple packinghouse facilities mostly during a 2-year environmental survey performed between 2016 and 2017 by Simonetti et al. (2021). In this FPE, Lm isolates belonging to IVb-v1, counted for more than 90% of all isolates and spread across the facility mostly contaminating floors and other NFCSs and persisting throughout the sampling year (Chen et al., 2022). These results were very similar to those obtained in our study and seemed to indicate that once strains belonging to this uncommon serogroup are introduced into an FPE, they are able to spread and persist mainly on NFCSs such as drains, floors, and cleaning materials.

Lm IVb-v1 strains have been identified among the isolates from human cases of listeriosis occurred in France, Brazil, Switzerland, and United States (Leclercq et al., 2011; Lee et al., 2012). These strains were responsible for maternal-neonatal forms, infection of central nervous system, and bacteremia. Also in Italy, a human case of listeriosis, caused by a IVb-v1 strain in 2016 was reported by Scaltriti et al. (2020).

All the IVb-v1 strains previously isolated worldwide, from both food and humans, belonged to four main STs and in particular to ST218, ST240, ST382, and ST554 (Chen et al., 2017, 2022; Kim et al., 2018; Yang et al., 2020). In particular, all the Italian isolates reported before belonged to ST218 within the CC218.Very interestingly, the IVb-v1 strains described in this study presented a new MLST allelic profile also included in the CC218 and assigned as ST2801. As a future perspective, the evolutionary linkage between these STs isolated in Italy could be investigated.

In this study, the ST2801 was isolated from different environmental surfaces, mostly NFCS, representing the main source of environmental contamination in the FPE. Most of these NFCS, represented ideal niches since they were difficult to clean and sanitize because of inherent inaccessibility (e.g., drains), harborage sites (e.g., porous wall-floor connections), wear surfaces (e.g., door seals), and cleaning tools (e.g., water pullets). On some of these NFCS, such as the wall floor connection of a cold room, the wheels of meat trolleys, the door handle and seal of another cold room, and the water puller of the washing area, the ST2801 was able to persist over the time even after cleaning and sanitation. Since the surfaces were all identified, these results were reported to the FBOs in order to increase their level of attention in terms of more accurate cleaning and sanitation or replacement of worn materials. The persistence of this ST probably indicated an efficient environmental adaptation also suggested by the presence of several genetic determinants for stress response, as discussed below. In their previous study, Chen et al. (2022) also reported the isolation of *Listeria monocytogenes* IVb-v1 strains from several NFCS and their ability to persist in the FPE.

CgMLST analysis identified three main clusters from two different types (CT10448 and CT11418), genetically close to each other. The results of SNPs analysis confirmed the presence of the same three close-to-clonal groups.

In particular, cluster A was the most represented and was isolated during all the sampling sessions performed both during production and after sanitation, persisting in the plant from July 2020 to September 2021. The same persistence period was observed for the smaller cluster B, which, however, unlike cluster A, was not isolated during the intermediate Production 2. Cluster C, instead, was isolated during Production 1 and Production 2 but not during Production 3. These three near-clonal clusters were found to be coexistent in the meat-producing plant until Production 2, while during Production 3, only cluster A and cluster B were isolated. As observed by Knudsen et al. (2017) and Chiaverini et al. (2021), the coexistence of closely related clusters over the time could be the result of a different evolution path starting from a common ancestor first introduced in the plant and/or the repetitive reintroduction of closely related clones probably by raw materials.

All the ST2801 strains carried several determinants for different heavy metals resistance, stress response, and multidrugs efflux pumps with no significant differences among the clusters.

Genes for heavy metals resistance included *czcD* encoding a cation diffusion facilitator protein family transporter reducing Cd^2+^, Zn^2+^, and Co^2+^ accumulation in the cytoplasm (Osman et al., 2021), *arsB* and *arsC* encoding an ATPase and an arsenate reductase involved in arsenic resistance (Parsons et al., 2018), the *csoR-copA-copZ* copper resistance operon (Corbett et al., 2011), and *zosA* for Zn(II) uptake also contributing to oxidative stress resistance (Gaballa and Helmann, 2002). For tolerance to acid stress, in addition to the *gadB-gadC* operon, for a glutamate decarboxylase and a glutamate/GABS antiporter (Cotter et al., 2005; Liu et al., 2019), all the studied strains presented *gadD*, for a homologous decarboxylase. This last gene was the only one detected within the SSI-1 (Ryan et al., 2010), while no determinants belonging to the SSI-2 were found.

Several genes associated with monovalent cation/proton antiporters were found in all the strains and included the multiple resistance and pH operon *mrpABCDEFG* encoding for Na^+^/H^+^ antiporters and *mdrP* for a Na^+^ Li^+^ K^+^/H^+^ antiporter, both responsible for sodium and alkali resistance (Abdel-Motaal et al., 2018; Fang et al., 2018; Xu et al., 2018; Yan et al., 2022).

The *crcB* gene, associated with the riboswitch responsible for tolerance to the anion fluoride, was also detected in the studied genomes (Baker et al., 2012; Johnston and Strobel, 2020; Chellaiah et al., 2021).

Different multidrug efflux pumps (*emrB*, *emrD*, *emrY*, *bmrA*, *bmr3*, *norM*, *mepA*, *mdrl*, and *lde*) and multidrug ABC transporters (*ybhF*, *ybhS*, *yheI*, and *yheH*) were detected in all the strains (Torres et al., 2009; Slipski et al., 2018; Sharma et al., 2019; Cherifi et al., 2020; Feng et al., 2020; Matereke and Okoh, 2020; Zhang et al., 2020). Many of these genomic features are known to be involved in tolerance to disinfectants, including QAC, largely used in the food industry and specifically in the meat-producing plant studied (Slipski et al., 2018; Sharma et al., 2019; Cherifi et al., 2020; Matereke and Okoh, 2020; Zhang et al., 2020).

Although all the ST2801 strains mostly exhibited the same genetic pattern associated with environmental adaptations, there were some differences between the plasmid-carrying strains and those without. In more detail, two different Listeria plasmids were carried by 54 strains; in 53 of them pLI100 (Glaser et al., 2001; Kuenne et al., 2010) and the j1776 plasmid (Chiaverini et al., 2021; Mafuna et al., 2021; Maggio et al., 2021) were detected, while in one of them, the pLM33 (Kuenne et al., 2010) was found in addition to pLI100. Only the strains carrying these plasmids presented *cadA* and *cadC* for cadmium resistance (Parsons et al., 2018), the *mco* gene encoding a multicopper oxidase (MCO), and the additional Na+/H+-K+ antiporter GerN (Southworth et al., 2001; Wu et al., 2020). This finding suggests that the above determinants were carried on these plasmids. We used the nucleotide Basic Local Alignment Search Tool (BLASTn) to verify the alignment between the sequences of *cadA*, *cadC*, *mco*, and *gerN* as annotated by Prokka in the studied strains, and the ones found in the GenBank file of each plasmid on NCBI. A 100% score for coverage and identity was observed between the *cadA* gene carried by the strains and the one present in both pLI100 and j1776 plasmids. The same result was obtained for the *cadC* and *gerN* carried by the strains compared with the respective genes carried by pLI100. A maximum alignment score was also found comparing the *mco* gene with the homologous gene carried by the j1776 plasmid. All these findings were in agreement with previous authors reporting these plasmids as carrying determinants for cadmium, arsenic and copper resistance (Canchaya et al., 2010; Parsons et al., 2018; Chiaverini et al., 2021; Lachtara et al., 2021; Schmitz-Esser et al., 2021). Moreover, according to several studies, the specific plasmids found in the strains of this new ST are highly conserved between Lm strains on a large scale and across other STs, suggesting that they provide important advantages for survival in food and FPEs (Canchaya et al., 2010; Parsons et al., 2018; Chiaverini et al., 2021; Guidi et al., 2021; Lachtara et al., 2021; Maggio et al., 2021; Schmitz-Esser et al., 2021).

Moreover, the coexistence of Lm strains carrying and not carrying plasmids, even belonging to the same genetic cluster, indicated horizontal transfer dynamics within the microbial population studied, most probably driven by selective pressure.

Although not presenting a Premature Stop Codon Mutation in the *inlA* gene nor the SSI-1, both associated with an increased production of biofilm, as reported in previous studies (Keeney et al., 2018; Upham et al., 2019; Mahoney et al., 2022), all the strains carried several genetic markers for biofilm production. In particular *luxS*, for a S-ribosylhomocysteinase, *recO* encoding the DNA repair protein RecO and lmo2504 for a cell wall-binding protein were found in all the genomes (Pasquali et al., 2018; Gorski et al., 2022). The *lmo0673* gene, encoding an hypothetical protein and reported as marker of biofilm production by Pasquali et al. (2018), was also found. These findings indicated the potential of biofilm formation of these strains. Regardless the effective amount of biofilm produced, if it is formed in niches that are difficult to reach during sanitation procedures, it can represent a persistent source of contamination. This could be another factor responsible for the survival of these ST2801 strains to disinfection.

Concerning the virulence profile, all the ST2801 strains presented the same identical pattern consistent with belonging to a IVb serogroup, albeit atypical and presenting hypervirulence features. Indeed, all the strains carried a full length *inlA*, encoding for a functional inlA, which is one of the essential virulence factors for Lm to cross the intestinal barrier. Other members of the internalin gene family were present in the strains, including *inlC*, *inlD*, *inlE*, *inlF*, *inlH*, *inlJ*, and *inlK*, which were known to contribute to the pathogenicity of the pathogen with different mechanisms (Su, 2019; Maćkiw et al., 2021). The *aut_IVb*, *gltA*, and *gltB* genes were also important virulence markers. These genes are involved in invasion and teichoic acid biosynthesis respectively, in serogroup IVb isolates, including IVb-v1, and are absent in other Lm (Moura et al., 2017).

The virulence profile of these ST2801 strains also included the additional LIPI-3, encoding a biosynthetic cluster involved in the production of listeriolysin S (LLS), a haemolytic, and cytotoxic factor conferring a greater virulence to Lm (Su, 2019; de Tavares et al., 2020). This finding was in agreement with previous studies reporting the presence of LIPI-3 in atypical IVb-v1 strains (Moura et al., 2017; de Tavares et al., 2020).

All these findings indicated this new ST, as presenting several determinants for stress response and environmental adaptation, probably responsible for its persistence in the meat-producing plant, together with virulence features. The isolation of a new MLST type with a large pattern of stress adaptation genes and able to persist over a year in the same FPE, also surviving cleaning and sanitation, is considered noteworthy.

## Conclusion

In this study, we discovered a new MLST allelic profile of Lm designed as ST2801, never reported before, and belonging to the atypical serogroup IVb-v1. Moreover, only one study existing before ours witnessed the isolation of IV-v1 Lm strains from FPEs and their ability to persist over time.

The isolation of this new ST occurred during an intensive environmental sampling plan for Lm performed in a pork meat-producing plant of Central Italy, during production and, for positive surfaces, also after cleaning sanitation. We found widespread circulation of the ST2801 in the studied environment, where it was able to persist over a year and survive sanitation. Based on genomic characterization results, this new ST presents several determinants for stress response, environmental adaptation, and biofilm production, probably responsible for its persistence in the meat-producing plant, together with virulence features.

The isolation of a new ST with a large pattern of stress adaptation genes and the ability to persist in the same FPE for a year is an important contribution to deepen the current knowledge on the uncommon IVb-v1 and in general on genomic diversity of Lm. At the same time, the sampling approach adopted allowed us to provide specific recommendations to the FBO in order to improve the control of the pathogen, minimizing the risk of food contamination. All these findings pointed out how the application of intensive environmental sampling plans, which are specific to each FPE, would ensure improved surveillance and would provide the opportunity to increase knowledge about Lm.

## Data Availability Statement

The datasets presented in this study can be found in online repositories. The names of the repository/repositories and accession number(s) can be found below: https://www.ncbi.nlm.nih.gov/bioproject/PRJNA821663.

## Author Contributions

All authors listed have made a substantial, direct, and intellectual contribution to the work, and approved it for publication.

## Conflict of Interest

The authors declare that the research was conducted in the absence of any commercial or financial relationships that could be construed as a potential conflict of interest.

## Publisher’s Note

All claims expressed in this article are solely those of the authors and do not necessarily represent those of their affiliated organizations, or those of the publisher, the editors and the reviewers. Any product that may be evaluated in this article, or claim that may be made by its manufacturer, is not guaranteed or endorsed by the publisher.

## Footnotes

1
http://bigsdb.pasteur.fr/listeria

2
https://github.com/usadellab/Trimmomatic

3
https://github.com/s-andrews/FastQC

4
https://github.com/ablab/spades

5
https://github.com/ablab/quast

6
http://bigsdb.pasteur.fr/listeria

7
https://github.com/B-UMMI/chewBBACA

8
https://github.com/achtman-lab/GrapeTree

9
https://sourceforge.net/projects/ksnp/

10
https://itol.embl.de/

11
https://github.com/tseemann/prokka

12
https://cge.cbs.dtu.dk/services/PlasmidFinder/

## Supplementary Material

Supplementary Table 1 Environmental sampling schema. FCS, food contact surface; NFCS, nonfood contact surface.

Supplementary Table 2 IVb variant 1 Lm strains, environmental surfaces from which they have been isolated and sampling data.

Supplementary Table 3 SNP distance matrix.
